# EIF2A promotes cell survival during paclitaxel treatment in vitro and in vivo

**DOI:** 10.1111/jcmm.14469

**Published:** 2019-06-18

**Authors:** Lin Chen, Jiang He, Jianhua Zhou, Zhi Xiao, Nianhua Ding, Yumei Duan, Wenzheng Li, Lun‐Quan Sun

**Affiliations:** ^1^ Center for Molecular Medicine Xiangya Hospital, Collaborative Innovation Center for Cancer Medicine, Central South University Changsha Hunan P.R. China; ^2^ Key Laboratory of Molecular Radiation Oncology Changsha Hunan P.R. China; ^3^ Department of Pathology Xiangya Hospital, Central South University Changsha Hunan P.R. China; ^4^ Department of Breast Surgery Xiangya Hospital, Central South University Changsha Hunan P.R. China; ^5^ Department of Radiology Xiangya Hospital, Central South University Changsha Hunan P.R. China

**Keywords:** chemotherapy, drug resistance, EIF2A, ISR, redox

## Abstract

The integrated stress response (ISR) is critical for cancer cell survival during stress stimuli and has been implicated in the resistance to cancer therapeutics, in which the mechanism, however, is poorly understood. Here, we showed that paclitaxel, the major chemotherapy drug for breast cancer, induced ISR and phosphorylated ser51 residue of EIF2S1 by EIF2AK3 and EIF2AK4. When exposed to paclitaxel, cancer cells activated the EIF2AK3/EIF2AK4‐pEIF2S1‐ATF4 axis and maintained redox homoeostasis by inducing expression of the major antioxidant enzymes HMOX1, SHMT2 and SLC7A11. Paclitaxel‐mediated cell death was significantly increased following loss of ISR or ATF4 expression. This sensitizing effect could be partially rescued by Trolox, a ROS scavenger. We demonstrated that the alternative initiation factor EIF2A was essential for cancer cell survival after paclitaxel‐mediated ISR both in vitro and in vivo. Moreover, patients with breast cancer exhibited higher ISR after chemotherapy, and the elevated mRNA levels of HMOX1, SHMT2 and EIF2A were correlated with poor prognosis. Collectively, our findings reveal a novel mechanism for paclitaxel resistance and suggest that targeting EIF2A combined with ISR agonist may be a potential treatment regimen to overcome drug resistance for breast cancer.

## 1 INTRODUCTION

In breast cancer chemotherapy, resistance to the drugs inevitably occurs, leading to tumour recurrence and disease progression. While the molecular mechanisms of drug resistance for different chemotherapeutics have been widely explored, none of them could be fully applied to the clinical settings. Therefore, deciphering unknown mechanisms of the resistance may help in identifying new treatment strategies for breast cancer patients.

The integrated stress response (ISR) is a mechanism, by which cells adapt to the microenvironment changes induced by stress stimuli.^1^ The hallmark of ISR is the phosphorylation of Ser51 residue on a subunit of the eukaryotic initiation factor 2, eIF2apha, encoded by the gene EIF2S1. This phosphorylated EIF2S1 down‐regulates translation initiation at AUG start codons. Four kinases participate in these processes, which are activated by different stress stimuli: the haeme‐regulated inhibitor kinase (HRI, encoded by EIF2AK1), activated by haeme deficiency^2^; the interferon induced double‐stranded RNA‐dependent eIF2a kinase (PKR, encoded by EIF2AK2), activated by viral infection^3^; endoplasmic reticulum resident kinase (PERK, encoded by EIF2AK3), activated by endoplasmic reticulum stress^4^; and the general control non‐derepressible 2 (GCN2, encoded by EIF2AK4), activated by amino acid deprivation.^5^

During the occurrence and development of tumours, cancer cells need to cope with various internal and external stresses. ISR has been suggested as a protective process to hypoxia and nutrient deprivation, as it is up‐regulated in ischaemic regions of tumours.^6^, ^7^ ISR is also reported to be involved in tumour metastasis and EMT process^8^, ^9^ and required to adapt to high metabolic demand during oncogenic transformation.^10^ For example, EIF2AK3 activation has been shown to promote cell transformation in different tumour models.^11^, ^12^ However, whether ISR plays critical roles in therapeutic responses for cancer is still to be further investigated.

Phosphorylation of EIF2S1 impairs global translation. However, some RNAs, such as ATF4 mRNA, which harbours a unique 3′ UTR, are translated more efficiently under ISR.^13^ Recent findings indicate that ATF4 is a key transcription factor to maintain amino acid metabolic homoeostasis,^14^ redox balance^9^, ^15^ and autophagy flux.^16^ Because of these effects, ATF4 is closely related to tumour growth,^17^ metastasis^9^ and resistance to some chemotherapeutic agents.^18^, ^19^, ^20^ Alternative initiation factor EIF2A has been considered as important translation factor shaping the ISR.^4^, ^21^ EIF2A‐mediated initiation pathway, which includes uORF translation, sustains expression of particular proteins during the ISR. A recent study reveals that EIF2A is essential for tumourigenesis and progression, because tumours exhibit more ISR than normal tissue in tumourigenesis, during which EIF2A maintains efficient translation of many genes related to tumourigenesis.^22^, ^23^

Here, we focused on the role of ISR in response of breast cancer cells to chemotherapy. We found that the ISR was activated immediately following paclitaxel treatment, but not Adriamycin. Two kinases, EIF2AK3 and EIF2AK4, contributed to this response. Higher ISR could been induced in breast cancer patients after paclitaxel treatment. Mechanistically, we found that the EIF2AK3/EIF2AK4‐pEIF2S1‐ATF4 axis contributed to redox homoeostasis by transcriptionally regulating antioxidant genes, such as HMOX1, SHMT2 and SLC7A11, rather than autophagic factors in the cells treated with paclitaxel. Finally, we demonstrated that EIF2A promoted cell survival during paclitaxel treatment both in vitro and in vivo. The current study suggests that targeting the EIF2A‐mediated translation in combination with paclitaxel may present a potential new strategy for breast cancer treatment.

## 2 MATERIALS AND METHODS

### 2.1 Cell lines and transfections

MDA‐MB‐231 and BT‐549 cells were cultured in DMEM containing 10% foetal bovine serum (Gibco). All cell lines were obtained from the American Type Culture Collection. Cells were transfected with siRNAs using the DharmaFECT1 transfection reagent (Thermo Fisher Scientific), and transfected with plasmid using ViaFect Transfection Reagent (Promega).

### 2.2 Plasmid and siRNA

Plasmid expressing EIF2S1 was constructed using a base vector of pLV‐EF1α‐MCS‐IRES‐Bsd (Biosettia). ShRNA targeting EIF2A, GCTCTATCTTGCACAAGTA, was cloned into Tet‐PLKO‐puro (Addgene). ShRNA targeting EIF2S1 was cloned into pLKO.1 (Addgene). ShRNA sequences for EIF2S1 3′ UTR were: a#, GCAGGTAGTTTGTACCATTTA; b#, GCCAGAGAATAGATCAGTATT. Helper plasmids for lentiviral production are pMD2.G (Addgene) and psPAX2 (Addgene). SiRNAs were purchased from Genepharma and the sequences were as follows (5 'to 3'): for ATF4,a# GTGAGAAACTGGATAAGAA, b# GCCTAGGTCTCTTAGATGA; for EIF2A, a# GCTCTATCTTGCACAAGTA, b# GGTTAATAATGGATACAAA; for HSPA5, a# GGAGCGCATTGATACTAGA, b# CAGATGAAGCTGTAGCGTA; for EIF2AK1, a# GATTAAGGGTGCAACTAAA, b# CGAAGAATCTTCCGAAGAA; for EIF2AK2, a# GACGGAAAGACTTACGTTA, b# GGTGAAGG TAGATCAAAGA; for EIF2AK3, a# GATTCGCAAGACCTTCAAT, b# CGCGGCAGGTCATTAGTAA; for EIF2AK4, a# GGTCCAAGGAAGCACCAAA, b# GGATCCCTTTTGCAAGATA.

### 2.3 Antibodies and chemicals

Antibodies used in the study were: HSPA5 (Santa Cruz Biotechnology, sc‐376768); EIF2S1 (Santa Cruz Biotechnology, sc‐133132); ATF4 (Cell Signaling Technology, 11815s); Phospho‐eIF2α (Ser51) (Cell Signaling Technology, 3597s); EIF2A (Proteintech, 11233‐1‐AP); LC3 (Proteintech, 12135‐1‐AP). Paclitaxel and Adriamycin were purchased from Sigma.

### 2.4 ROS measurements

Cells were incubated with 10 μmol/L DCF‐DA (Sigma‐Aldrich) at 37°C for 30 minutes and analysed by flow cytometry following the manufacturer's instruction.

### 2.5 Cell viability and apoptosis assay

Cell viability was also measured by CCK‐8 (Dojindo) following the manufacturer's instruction. All viability experiments were repeated in three independent experiments, and Student's *t* test was used for calculating statistical significance. Cell apoptosis was detected by PI and staining was carried out according to the Apoptosis Detection Kit (Biotool).

### 2.6 Real‐time quantitative PCR

RNA was extracted by TRIzol Reagent (Invitrogen). Reverse transcription was performed with PrimeScript™ RT reagent Kit (Takara). Real‐time PCR was done using iTaq Universal SYBR Green Supermix (Bio‐Rad) in CFX96 Touch™ Real‐Time PCR Detection System (Bio‐Rad). PCR primers (5'‐3') were: ATF3, F: CAGAGTGGGTCTTGGACCAG, R: AGTGACAATGGTAGCCAC GG; DDIT3, F: GCTCAGGAGGAAGAGGAGGA, R: TCCTGCTTGAGCCGTT CATT; PPP1R15A, F: GTATGGTGAGCGAGAGGCAA, R: TCCCGGTGTGATGGT GGATA; HMOX1, F: ACTCCCTGGAGATGACTCCC, R: TCTTGCACTTTGTTGCT GGC;SHMT2,F: GAGACCGAAGTGCCATCACA,R: AATCCTGGAGCTTGGCA GTC;SLC7A11, F: TTTTCTGAGCGGCTACTGGG, R: CAGCTGGTAGAGGAG TGTGC;EIF2AK1,F: GGAACTCATCGCAGAGACCA, R: CCCCCATCCTTTCC GTCATC; EIF2AK2, F: GTGGACCTCTACGCTTTGGG, R: TGGGCTTTTCTT CCACACAGT; EIF2AK3,F: TGGGACCAAGACCGTGAAAG, R: TCGTCACT ATCCCATTGGCG; EIF2AK4, F: ACATCGGGCAAACTCCTCAG, R: CCAGT GGCTGTTTCCAAAGC; GAPDH, F: GCCGTCTAGAAAAACCTGCC, R: AAAG TGGTCGTTGAGGGCAA.

### 2.7 Immunohistochemistry

Paraffin‐embedded tissue slides were obtained from the Pathology Department of Xiangya Hospital of Central South University and the use of the samples was approved by Human Ethic Committee of Xiangya Hospital. Immunohistochemistry was performed with antibodies against p‐EIF2S1 and EIF2A. Stained slides were assessed and quantified in a blinded manner by the qualified pathologists. Paired *t* test was used for calculating statistical significance.

### 2.8 Xenograft model

All animal procedures were approved by the Animal Ethics Committee of Central South University. 3 × 10^6^ MDA‐MB‐231 cells resuspended in 100 μL of Matrigel (Corning) were subcutaneously injected into 6‐week old nude mice. The mice were fed with doxycycline water (1000 mg/L) when the tumours reached a size of around 60 mm^3^. Paclitaxel (20 mg/kg) was administered by intraperitoneal injection twice a week when the tumours were about 100 mm^3^. Tumours were measured every 3 days.

## 3 RESULTS

### 3.1 Paclitaxel‐induced ISR in breast cancer cells

**Figure 1:**
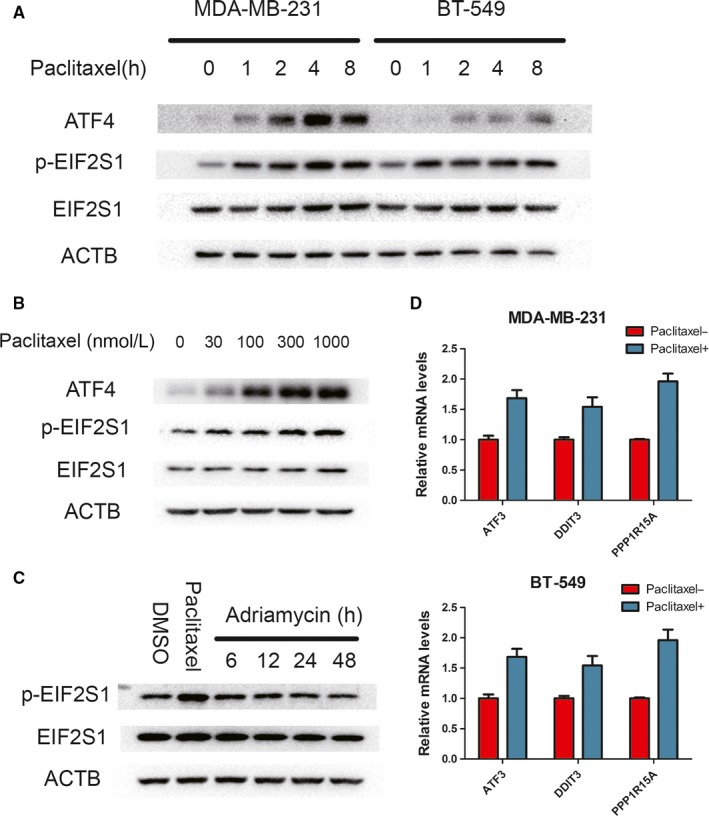
Integrated stress response (ISR) induction by paclitaxel, but not Adriamycin. A, MDA‐MB‐231 and BT‐549 were treated with paclitaxel (100 nmol/L) for indicated hours. Western blots performed with indicated antibodies. B, MDA‐MB‐231 cell line was incubated different concentrations of paclitaxel for 2 h. Cell lysates were immunoblotted with indicated antibodies. C, MDA‐MB‐231 cell line was incubated with 1 μmol/L Adriamycin for indicated hours or 100 nmol/L paclitaxel for 2 h. WB was performed with indicated antibodies. D, MDA‐MB‐231 and BT‐549 were incubated with 100 nmol/L paclitaxel for 4 hours. mRNA levels for ATF3, DDIT3 and PPP1R15A relative to GAPDH were measured by RT‐PCR

Paclitaxel and Adriamycin are the main drugs used in breast cancer neoadjuvant chemotherapy.^24^, ^25^ To examine the effect of these drugs on ISR induction, we treated breast cancer cell lines MDA‐MB‐231 and BT‐549 with these drugs and detected the phosphorylation of Ser51 residue on EIF2S1 and its downstream ATF4 expression.^1^ Western blotting showed that these two hallmarks of ISR could be robustly induced following paclitaxel treatment within only 1 hour (Figure 1A). The ISR became severe with increase in the concentration of paclitaxel (Figure 1B). Meanwhile, the mRNA levels of ATF4 transcriptional targets, ATF3, DDIT3 and PPP1R15A,^1^ were also up‐regulated 4 hours after treatment (Figure 1D). However, no significant change in ISR was detected following Adriamycin treatment (Figure 1C). These results suggest that chemotherapeutics‐induced ISR can be a drug‐type‐dependent response.

### 3.2 EIF2AK3 and EIF2AK4 contribute to paclitaxel‐mediated ISR

**Figure 2:**
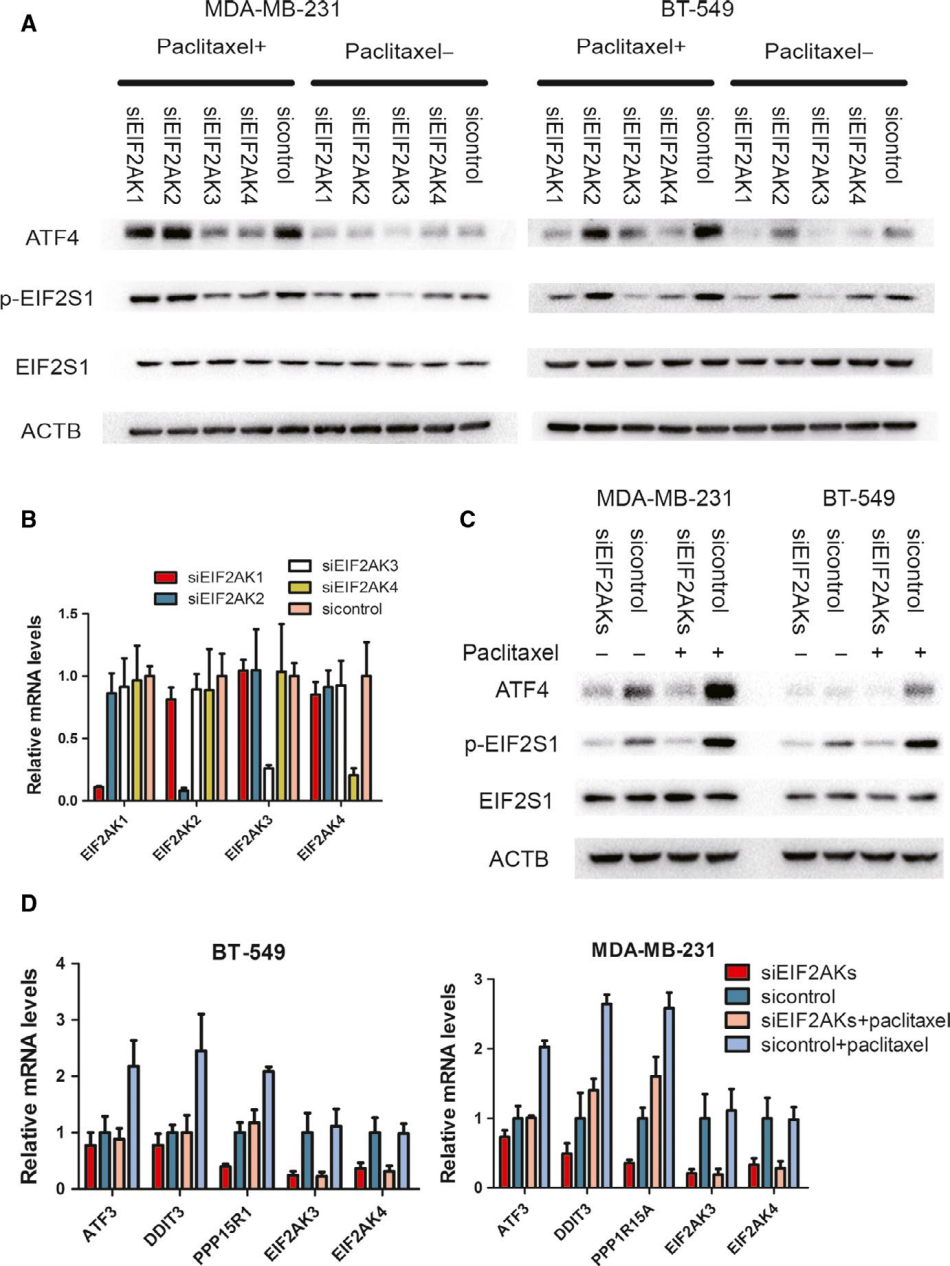
ISR induced by paclitaxel is mediated by EIF2AK3 and EIF2AK4. A, MDA‐MB‐231 and BT‐549 transfected with indicated siRNA were incubated with 100 nmol/L paclitaxel for 1 h. Cell lysates were immunoblotted with indicated antibodies. B, MDA‐MB‐231 was transfected with indicated siRNA. The mRNA levels relative to GAPDH were measured by RT‐PCR. C, MDA‐MB‐231 and BT‐549 transfected with indicated siRNA (siEIF2AKs indicate the mix of siEIF2AK3 and EIF2AK4) were incubated with 100 nmol/L paclitaxel for 4 h. Western blot was performed with indicated antibodies. D, MDA‐MB‐231 and BT‐549 transfected with indicated siRNA (siEIF2AKs indicate the mix of siEIF2AK3 and EIF2AK4) were incubated with 100 nM paclitaxel for 4 hours. RT‐PCR was performed with indicated genes

Next, we attempted to identify which kinases contribute to the paclitaxel‐induced ISR. siRNAs targeting all four kinases were used to inhibit ISR 1 hour after paclitaxel treatment.^26^ The screening showed that both EIF2AK3 (PERK) and EIF2AK4 (GCN2) could efficiently cause paclitaxel‐induced EIF2S1 phosphorylation, as well as downstream ATF4 expression in MDA‐MB‐231 and BT‐549 cell lines (Figure 2A,B). To further confirm this observation, we knocked down EIF2AKs (EIF2AK3 and EIF2AK4) and measured the ISR‐related markers. The phosphorylation of EIF2S1 and ATF4 expressions was almost completely abolished as well as for the mRNA levels of ATF3, DDIT3 and PPP1R15A when EIF2AK3 and EIF2AK4 were knocked down, in both MDA‐MB‐231 and BT‐549 cell lines (Figure 2C‐D). Therefore, the ISR in breast cancer cells after paclitaxel treatment may be induced by a co‐ordinated effect conferred by EIF2AK3 and EIF2AK4.

### 3.3 Loss of ISR increased paclitaxel‐mediated cell death

**Figure 3:**
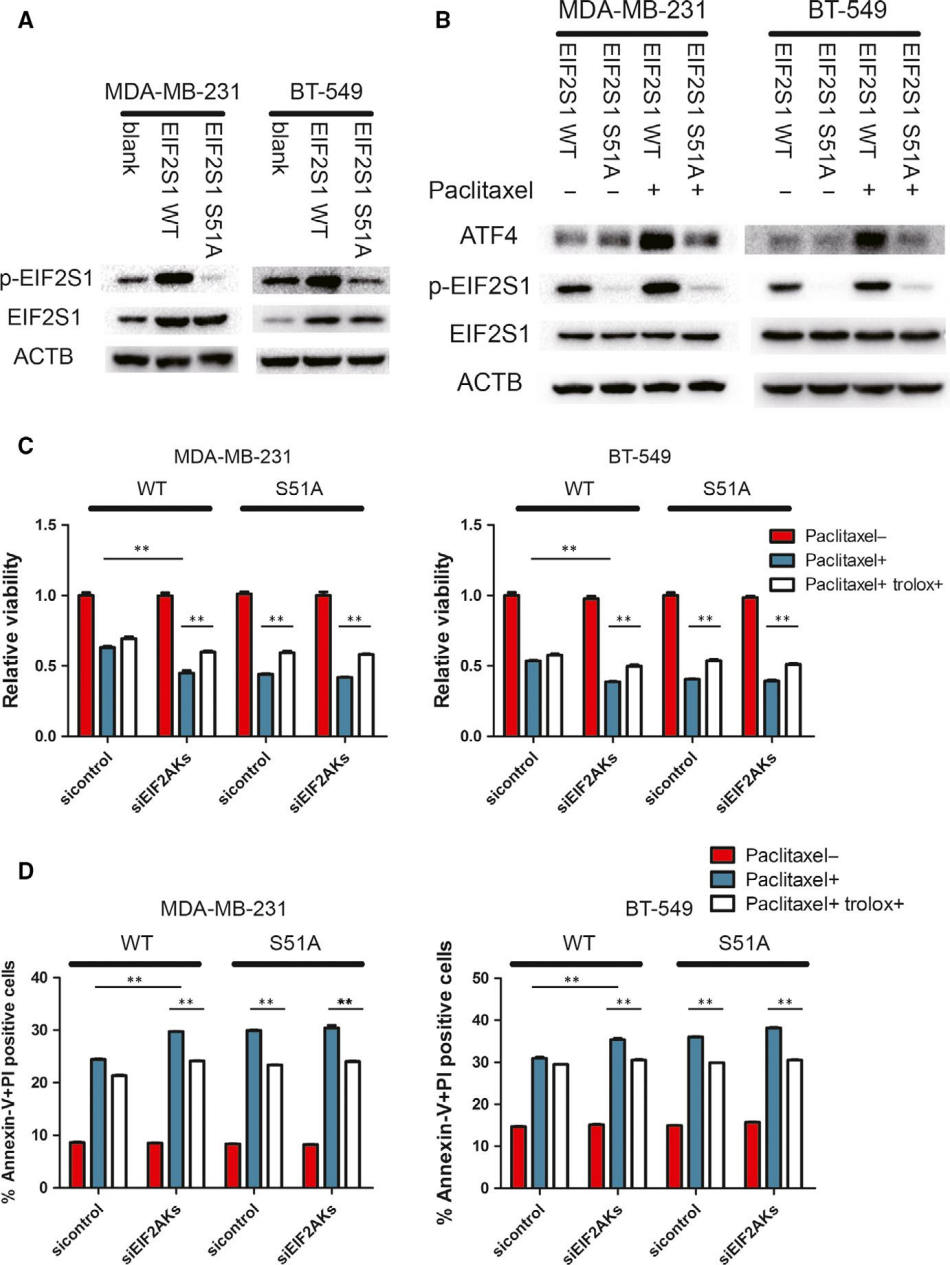
Induction of the ISR is critical for resistance to paclitaxel. A, Immunoblots of lysates from blank (without transfected) and EIF2S1 WT (shEIF2S1 + widetype EIF2S1) and EIF2S1 S51A (shEIF2S1 + S51A mutated EIF2S1). B, Indicated cells were incubated with 100 nmol/L paclitaxel for 4 hours and Western blots were performed. C, Indicated cells were incubated with 100 nmol/L paclitaxel and 50 μmol/L Trolox for 48 h. Cell viability was performed by CCK‐8 assay. Percentage of cell survival is represented as mean ± SD from three independent experiments (n = 3, mean ± SD). **P < 0.01, Student's *t* test. D, Indicated cells stained with Annexin‐V and PI, and analysed by FACS. Bars indicate mean values ± SD of three experiments. **P < 0.01

**Figure 4:**
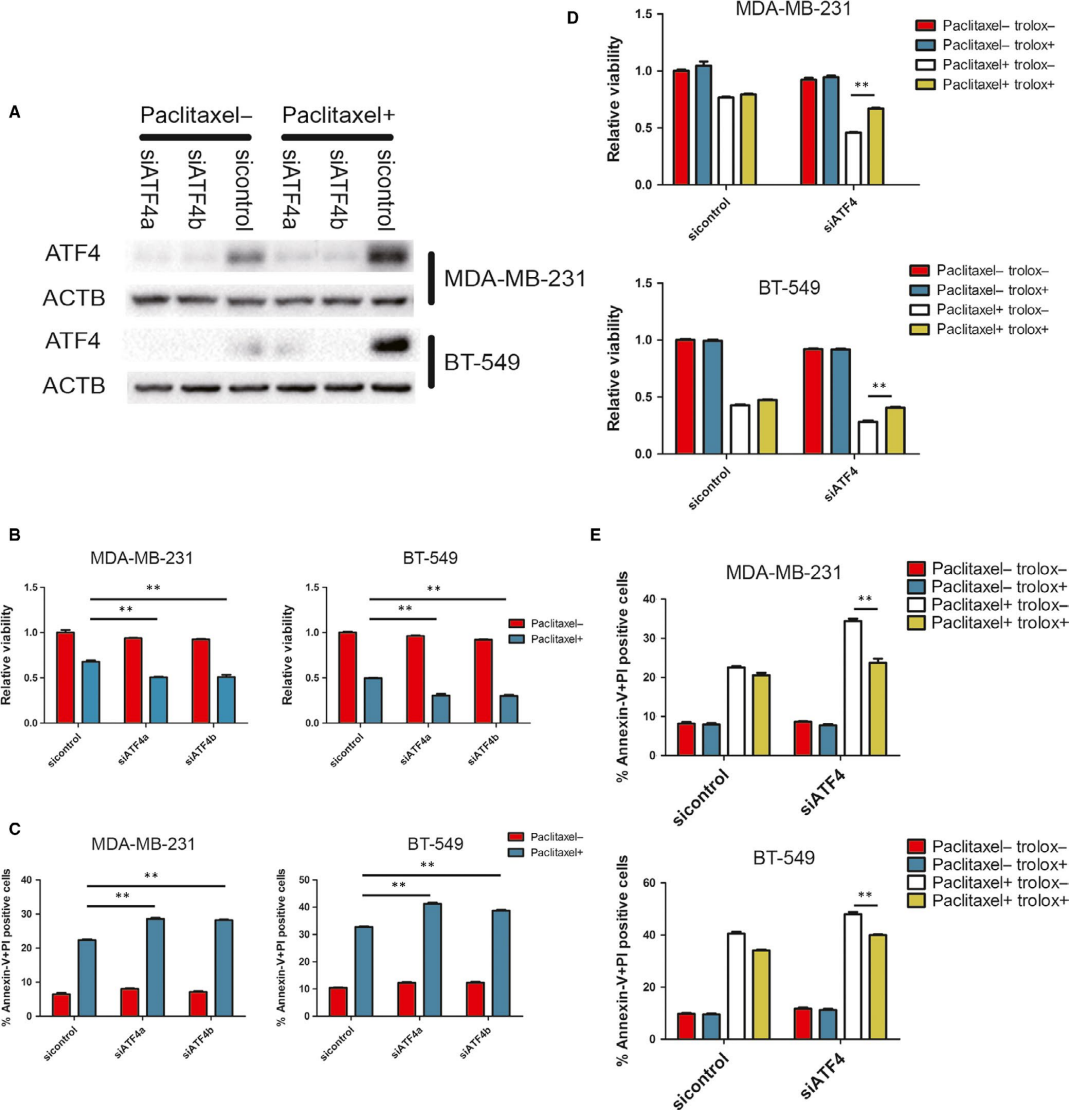
ATF4 confers resistance to paclitaxel. A, MDA‐MB‐231 and BT‐549 were transfected with indicated siRNA and incubated with 100 nmol/L paclitaxel for 4 h. Western blots were performed with indicated antibodies. B, Indicated cells were incubated with 100 nmol/L paclitaxel for 48 h. Cell viability was measured by CCK‐8 assay. Percentage of cell survival is represented as mean ± SD from three independent experiments (n = 3, mean ± SD). **P < 0.01, Student's *t* test. C, Indicated cells stained with Annexin‐V and PI, and analysed by FACS. Bars indicate mean values ± SD of three experiments. **P < 0.01. D, Indicated cells were incubated with 100 nmol/L paclitaxel and 50 μmol/L Trolox for 48 h. Cell viability was measured by CCK‐8 assay. Percentage of cell survival is represented as mean ± SD from three independent experiments (n = 3, mean ± SD). **P < 0.01, Student's *t* test. E, Indicated cells were incubated with 100 nmol/L paclitaxel and 50 μmol/L Trolox for 48 h and stained with Annexin‐V and PI, and analysed by FACS. Bars indicate mean values ± SD of three experiments. **P < 0.01

To further validate the key role of EIF2S1 phosphorylation in ISR induced by paclitaxel, we established cell lines expressing either the wild‐type EIF2S1 or S51A mutated EIF2S by first knocking down endogenous EIF2S1 with shRNA targeting at the 3′ UTR of EIF2S1 mRNA and then exogenously re‐expressing wild‐type EIF2S1 or S51A mutated EIF2S1.^27^, ^28^ As expected, ISR could not be induced in the cells expressing S51A mutant (Figure 3A‐B). To determine the role of ISR in cell fate, we treated WT and S51A cells with paclitaxel respectively. EIF2S1 S51A mutation decreased cell viability compared to wild‐type EIF2S1. Moreover, EIF2AKs knockdown could not further influence cell viability when EIF2S1 was mutated, in comparison with the EIF2S1 WT cells, which showed an obvious decrease in viability when knocking down EIF2AKs (Figure 3C). Thus, the EIF2AKs‐EIF2S1 axis regulated the cell fate after paclitaxel treatment. Furthermore, Fluorescence‐activated cell sorting (FACS) analysis of breast cancer cells treated with paclitaxel also revealed that knockdown of EIF2AKs sensitized cells to paclitaxel‐induced apoptosis, and these effects mainly through EIF2S1 phosphorylation (Figure 3D). When the role of ATF4 in facilitating resistance to paclitaxel was further determined, it was found that knockdown of ATF4 expression by siRNAs in both cell lines resulted in lower viability and higher levels of apoptosis rate (Figure 4A‐C). These results substantiate the importance of ISR in maintaining cell survival under paclitaxel treatment.

### 3.4 ISR‐dependent redox homoeostasis protected cancer cell from paclitaxel‐mediated cell death

**Figure 5:**
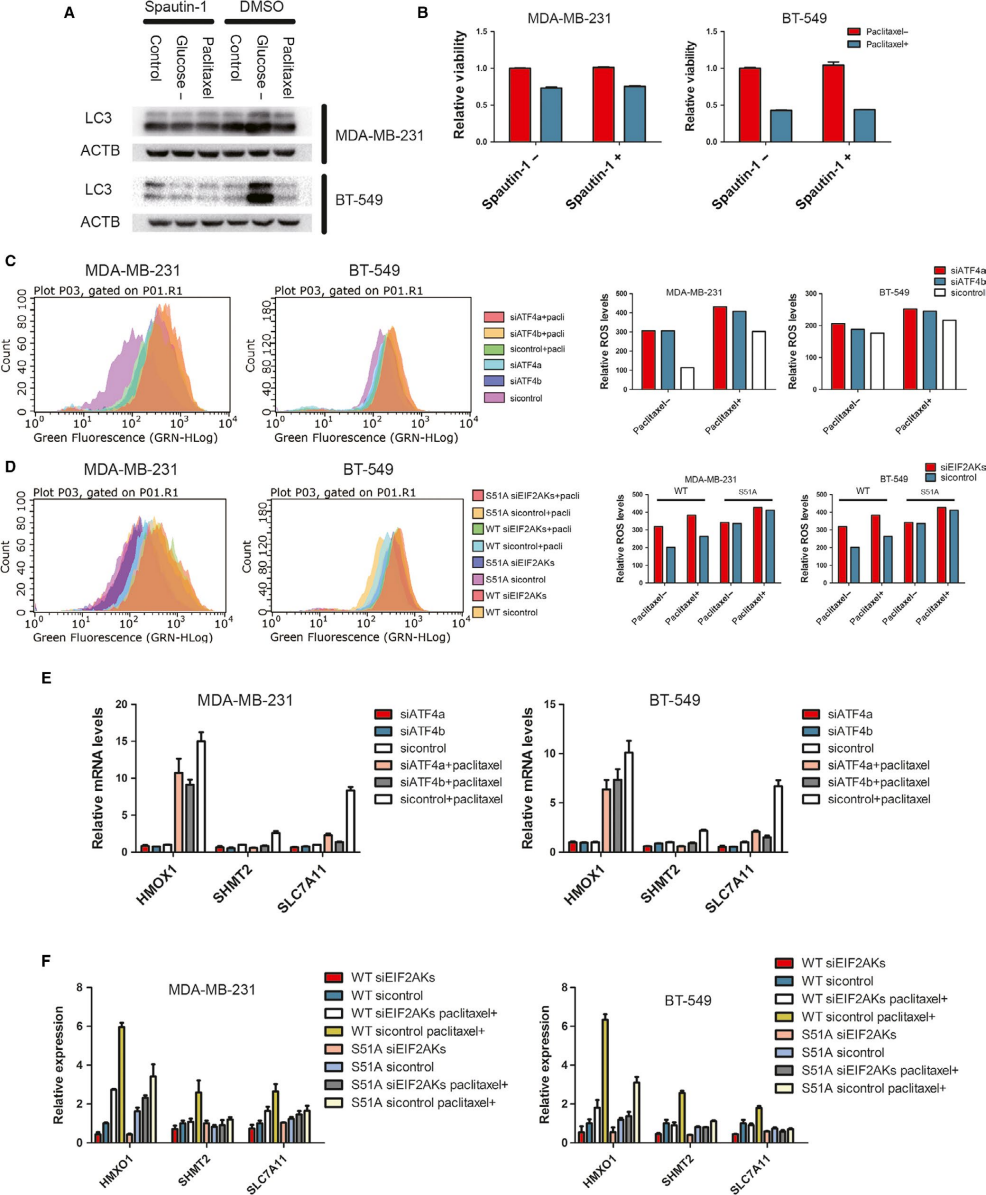
Loss of ISR induces oxidative stress after paclitaxel treatment. A, MDA‐MB‐231 and BT‐549 were treated as indicated. Spautin‐1 (5 μmol/L) was used to inhibit autophagy. Western blot was performed with indicated antibodies. B, Indicated cells were incubated with 100 nmol/L paclitaxel and 5 μmol/L spautin‐1 for 48 h. Cell viability was measured by CCK‐8. Percentage of cell survival is represented as mean ± SD from three independent experiments (n = 3, mean ± SD). **P < 0.01, Student's *t* test. C and D, Left: Indicated cells were incubated with 100 nmol/L paclitaxel for 24 h. ROS was labelled by DCF‐DA (10 μmol/L), and analysed by FACS. Right: panels show mean fluorescence intensity that reflects relative ROS levels. E and F, mRNA levels for HMOX1, SHMT2, and SLC7A11 relative to GAPDH were measured by RT‐PCR in indicated cells transfected with indicated siRNA and incubated with paclitaxel (100 nmol/L) for 16 h. Data are represented as mean fold change compared with attached cultures for three independent experiments (n = 3, mean ± SD). **P < 0.01, Student's *t* test

Previous studies showed that IRS and ATF4 were essential for stress‐induced autophagy.^1^, ^5^, ^16^, ^29^ Many commonly used chemotherapeutic drugs could activate autophagy, which suppressed the cytotoxic effects of the drugs in most cases.^30^ To investigate the role of autophagy in the efficacy of paclitaxel, we first analysed whether paclitaxel could induce an increase in autophagy. Western blotting showed that paclitaxel treatment had no substantial effect on processing of LC3BI to LC3BII, compared to glucose deprivation, in both MDA‐MB‐231 and BT‐549 cells (Figure 5A). Furthermore, autophagy inhibitor, spautin‐1, failed to further decrease cell viability after paclitaxel treatment (Figure 5B). These results largely ruled out the effect of the IRS‐related autophagy on paclitaxel‐mediated cell death in vitro.

Paclitaxel has been shown to have a bystander effect on cancer cells mediated by ROS.^31^ To examine if redox homoeostasis is involved in protection of cancer cells from paclitaxel‐induced cell death, we first detected the ROS level through small‐molecule fluorescent probe (DCF‐DA). Paclitaxel treatment increased the ROS levels in both MDA‐MB‐231 and BT‐549 (Figure 5C). Moreover, ATF4 participated in redox balance after paclitaxel treatment, as ROS levels further increased after ATF4 knockdown (Figure 5C). More importantly, ROS scavenger, Trolox, partially rescued the paclitaxel‐mediated cell death after ATF4 knockdown (Figure 4D‐E).

Next, we examined the role of ISR in redox homoeostasis following paclitaxel treatment. EIF2S1 S51A cells had higher ROS level than EIF2S1 WT following paclitaxel treatment, indicating that EIF2AKs‐EIF2S1 axis participated in redox homoeostasis after paclitaxel treatment. In contrast to EIF2S1 WT cell lines, knockdown of EIF2AKs could not increase ROS levels in EIF2S1 S51A cell lines after paclitaxel treatment (Figure 5D). In the subsequent cell viability and cell apoptosis assays, Trolox partially rescued cell death from ISR loss (Figure 3C,D).

To further validate the above results, we analysed the expression levels of antioxidant genes, including HMOX1,^9^, ^32^ SHMT2^15^, ^33^ and SLC7A11.^34^, ^35^, ^36^ The transcript levels of these genes were markedly increased 16 hours after paclitaxel treatment and knockdown of ATF4 down‐regulated their mRNA levels (Figure 5E). Importantly, loss of ISR also significantly reduced the transcription of antioxidant genes (Figure 5F).

Taking together, these results strongly suggest that ISR and ATF4 expression leads to up‐regulation of antioxidant genes to deal with the increased oxidative stress and promote cancer cells survival after paclitaxel treatment.

### 3.5 EIF2A is essential for paclitaxel sensitivity during paclitaxel‐mediated ISR

**Figure 6:**
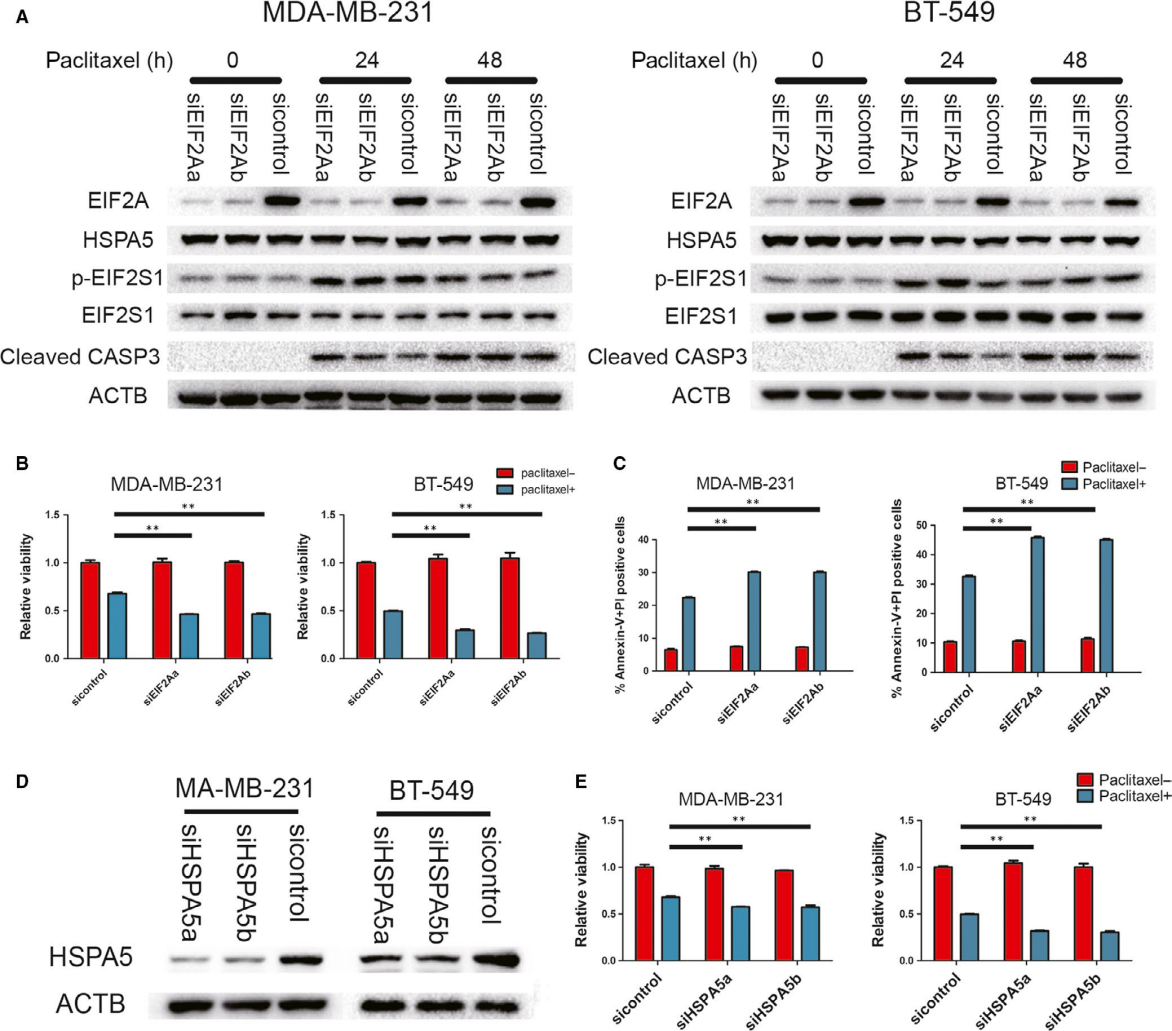
EIF2A promotes cell survival during paclitaxel treatment in vitro. A, Indicated cells were transfected with EIF2A siRNA, and followed by paclitaxel treatment. Cells were lysed at indicated time points for Western blot assays of EIF2A, HSPA5, p‐EIF2S1, EIF2S1 and cleaved caspase 3. B and E, Indicated cells transfected with indicated siRNA were incubated with 100 nmol/L paclitaxel for 48 h. Cell viability was measured by CCK‐8 assay. Percentage of cell survival is represented as mean ± SD from 3 independent experiments (n = 3, mean ± SD). **P < 0.01, Student's *t* test. C, indicated cells stained with Annexin‐V and PI, and analysed by FACS. Bars indicate mean values ± SD of three experiments. **P < 0.01. D, MDA‐MB‐231 and BT‐549 transfected with indicated siRNA. Western blots were performed with indicated antibodies

Recent studies emphasized the importance of EIF2A, the alternative initiation factor, on tumour initiation.^23^ EIF2A maintains expression of particular proteins when conventional translation was weakened 22, 37. Therefore, we wonder whether EIF2A‐mediated translation confers resistance to paclitaxel treatment during ISR. Knockdown of EIF2A impaired the expression of HSPA5, an EIF2A‐regulated chaperone, after paclitaxel treatment (Figure 6A). This suggested EIF2A participated in translation control during paclitaxel‐related ISR. To test this effect on paclitaxel sensitivity, cell viability and apoptosis assays were performed. Cell viability was significantly decreased in EIF2A knockdown cells 48 hours after treatment (Figure 6B). Cell apoptosis rate and the level of cleaved caspase 3 were also in accordance with viability assay (Figure 6A,C). Considering HSPA5 is an essential chaperone for unfold protein response,^38^ we knocked down HSPA5 and cell viability was shown to be decreased in both MDA‐MB‐231 and BT‐549 cell lines (Figure 6D,E).

**Figure 7:**
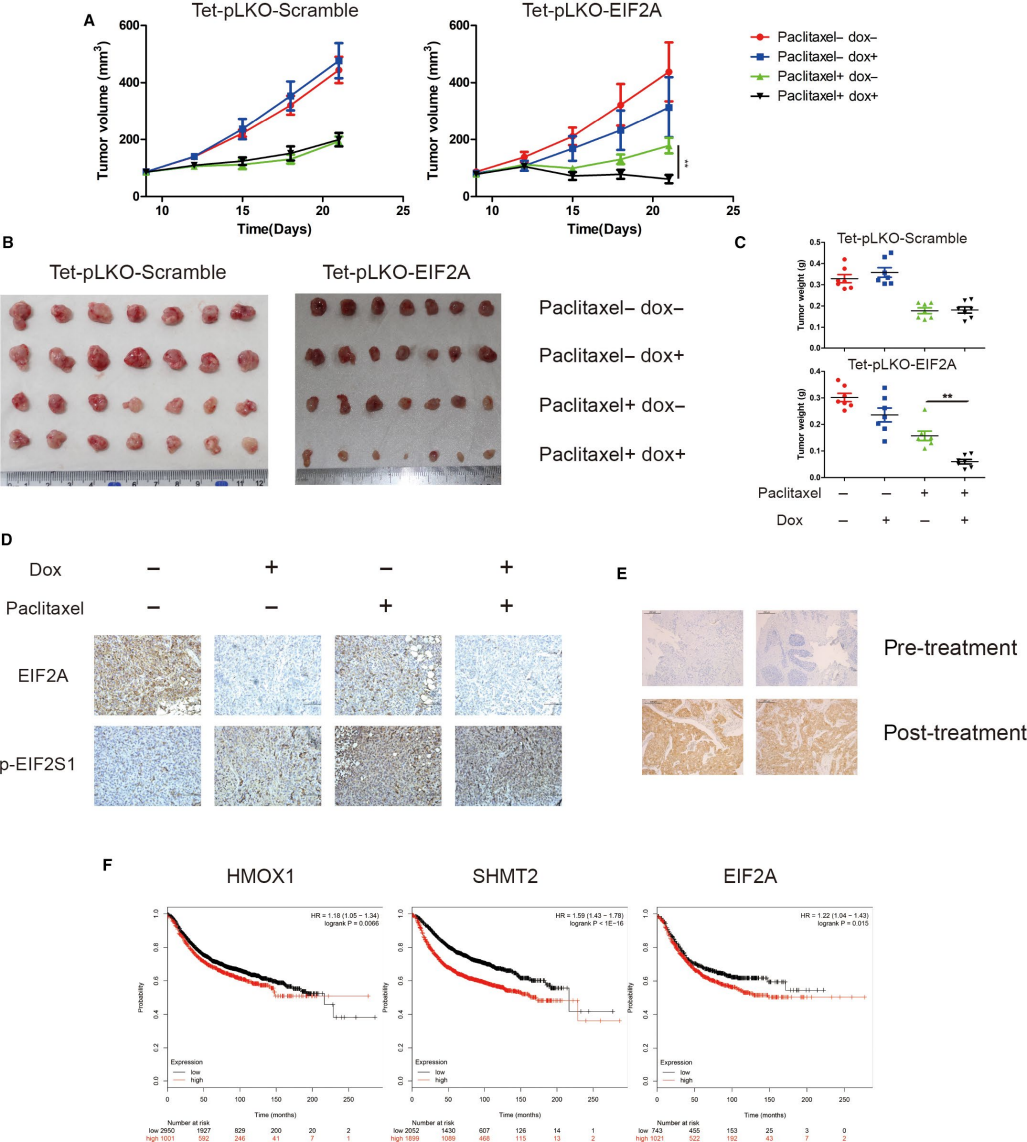
EIF2A promotes cell survival during paclitaxel treatment in vivo. A, B and C, Size and weight of xenograft tumours formed by MDA‐MB‐231 derivatives including a doxycycline‐inducible shRNA targeting EIF2A and scramble shRNA. Each group was treated as indicated. Data are mean ± SEM **P < 0.01. D, Immunohistochemical analyses of the expression of EIF2A and p‐EIF2S1 in xenograft tumours as described above. E, Immunohistochemical analyses of patient samples for p‐EIF2S1 levels in patients. Top: paracentesis specimens before treatment. Bottom: surgical specimens after paclitaxel‐based neoadjuvant chemotherapy. F, Immunohistochemical analysis was graded on a scale of 1‐3 according to staining intensity. Specimens form 30 patients participated in the statistic. Paired *t* test was used for calculating statistical significance. G, Probability of relapse‐free survival in 3955 breast cancer patients stratified on low (black) versus high (red) expression levels of indicated genes was obtained from Kaplan‐Meier Plotter/breast cancer (http://www.kmplot.com)

To determine the role of EIF2A in regulation of paclitaxel‐therapeutic efficacy in vivo, we grafted MDA‐MB‐231 derivative with a doxycycline‐inducible shRNA targeting EIF2A in nude mice. In mice treated with doxycycline drinking water to knock down EIF2A, paclitaxel was shown to be more efficacious in inhibiting tumour growth than in the controls (Figure 7A‐C). Immunohistochemical staining showed that the tumours treated with doxycycline exhibited lower expression of EIF2A and stronger phosphorylated EIF2S1 than that in the controls (Figure 7D). In order to rule out the effect of doxycycline itself on tumour growth, the doxycycline‐inducible scramble shRNA group was set up. Although paclitaxel could still suppress tumour growth, scramble shRNA expression induced by doxycycline could not further reduce tumour size (Figure 7A‐C).

These data suggest that during the paclitaxel‐mediated ISR, EIF2A selectively increases translational efficiency of certain genes and confers paclitaxel resistance.

### 3.6 ISR is induced in human breast cancer by paclitaxel treatment

Given our in vitro and in vivo observations on paclitaxel‐mediated ISR, we further validated this response in breast cancer patients. Through comparing the levels of EIF2S1 S51 phosphorylation in pre‐paclitaxel treatment breast cancer tissues to post‐paclitaxel treatment tissues by immunochemistry analysis, we found a significant increase of phosphorylated EIF2S1 in breast cancer samples following paclitaxel‐based neoadjuvant chemotherapy (Figure 7D‐E). In addition, analyses of publicly available data sets revealed that the mRNA levels of the ISR‐related antioxidant genes, HMOX1 and SHMT2, negatively correlated with relapse‐free survival of breast cancer patients. Notably, higher EIF2A mRNA levels correlated significantly with shorter relapse‐free survival (Figure 7F). Together, paclitaxel treatment could induce ISR to provide survival advantage for cancer in vivo.

## 4 DISCUSSION

Resistance to chemotherapy is one of the major problems in cancer therapy. Many mechanisms of resistance to ‘classical’ cytotoxic chemotherapies have been revealed, such as alternating drug targets, efflux of drugs and activating pro‐survival pathways.^39^ In this study, we explored the potential roles of ISR in mediating resistance to some chemotherapeutics for breast cancer cells. ISR is a conservative mechanism to sustain homoeostasis when suffering from stress. Depending on cell types, stress duration and intensity, and many other factors, the cells exhibit different outcomes of ISR.^1^, ^6^ Recent studies suggested that cancer cells experienced an impaired canonical translation and directed translational machinery to EIF2A‐dependent translation when encountering various microenvironmental stresses in tumourigenesis.^23^ Here, we show that paclitaxel induces ISR in breast cancer cells and patient samples. During the paclitaxel‐mediated ISR, EIF2A is essential for paclitaxel sensitivity and loss of ISR increases paclitaxel‐mediated cell death. Thus, ISR may represent a novel mechanism to protect breast cancer cells from paclitaxel‐mediated cell death.

Upon paclitaxel treatment, we identified EIF2AK3 and EIF2AK4 as the main kinases in regulating EIF2S1 phosphorylation, leading to IRS. However, this is not the case for Adriamycin, in which the treatment did not cause phosphorylation of EIF2S1. Microtubules are involved in regulating endoplasmic reticulum (ER) morphology and trafficking. Maintenance of ER homoeostasis tightly relies on the microtubule cytoskeleton.^40^, ^41^ Moreover, Brefeldin‐A, an inhibitor of ER‐to‐Golgi trafficking, could similarly induce ER stress.^42^ This reasonably explains why the microtubule stabilizer paclitaxel, but not Adriamycin, can cause ER stress‐induced ISR. In addition, EIF2AK4, a well‐known amino acid sensor, was found here to participate in the paclitaxel‐induced ISR. A recent study brought a hint that paclitaxel could induce degradation of glutamine carrier proteins and reduce glutamine uptake after long‐term administration of paclitaxel.^43^ Our study found that EIF2AK4 activated ISR within only 1 hour after paclitaxel treatment, which indicates that there may exist a more direct mechanism to achieve the level of ISR in the treated breast cancer cells.

In the course of cancer treatment. Cancer cells are frequently subject to heavily attack from ROS. ATF4 acts as a master transcription factor, which up‐regulates the genes responsible for metabolic reprograming, contributes to overwhelm aberrant oxidative stress in cancer.^15^, ^44^ GSH, as a powerful antioxidant and antidote, is transcriptionally regulated by ATF4 and also plays a role in resistance to many chemotherapeutics. High mRNA levels of the genes related to GSH synthesis have been shown to be associated with unfavourable clinical outcome in patients.^45^ Interestingly, paclitaxel has been shown to have a bystander effect mediated by ROS, which was released through enhancing the activity of NADPH oxidase associated with plasma membranes.^31^ In neuronal models, the microtubule network was related to oxidative stress through direct structural changes and protein‐protein interactions.^46^

Collectively, our data reveal an important role of ISR in resistance mechanisms for the drugs that affect microtubules, such as paclitaxel, suggesting that ISR is an important target in the treatment of cancers by pharmacological modulation. Our findings may also have important clinical implications and give a clue that EIF2A‐mediated translation during ISR may be a potential therapeutic target.

## CONFLICT OF INTEREST

We declare that we do not have any commercial or associative interests that represent a conflict of interest in connection with the work submitted.

## AUTHOR CONTRIBUTIONS

All authors participated in designing the concept of this manuscript. LC acquired the data and drafted the article. All authors finalized the paper and provided suggestions to improve it.
